# Optical coherence angiography for pre-treatment assessment and treatment monitoring following photodynamic therapy: a basal cell carcinoma patient study

**DOI:** 10.1038/s41598-019-55215-6

**Published:** 2019-12-10

**Authors:** E. V. Gubarkova, F. I. Feldchtein, E. V. Zagaynova, S. V. Gamayunov, M. A. Sirotkina, E. S. Sedova, S. S. Kuznetsov, A. A. Moiseev, L. A. Matveev, V. Y. Zaitsev, D. A. Karashtin, G. V. Gelikonov, L. Pires, A. Vitkin, N. D. Gladkova

**Affiliations:** 1 Privolzhsky Research Medical University, Minina Square 10/1, 603005 Nizhny Novgorod, Russia; 2 A. Tsyb Medical Radiological Research Center, Korolev Street 4, Obninsk, 249036 Kaluga region Russia; 3 N.A. Semashko Nizhny Novgorod Regional Clinical Hospital, Rodionova Street 190, 603093 Nizhny Novgorod, Russia; 4 Institute of Applied Physics Russian Academy of Science, Ulyanova Street 46, 603950 Nizhny Novgorod, Russia; 5 University of Toronto and University Health Network, 610 University Ave., Toronto, Ontario M5G 2M9 Canada

**Keywords:** Cancer therapy, Basal cell carcinoma

## Abstract

Microvascular networks of human basal cell carcinomas (BCC) and surrounding skin were assessed with optical coherence angiography (OCA) in conjunction with photodynamic therapy (PDT). OCA images were collected and analyzed in 31 lesions pre-treatment, and immediately/24 hours/3–12 months post-treatment. Pre-treatment OCA enabled differentiation between prevalent subtypes of BCC (nodular and superficial) and nodular-with-necrotic-core BCC subtypes with a diagnostic accuracy of 78%; this can facilitate more accurate biopsy reducing sampling error and better therapy regimen selection. Post-treatment OCA images at 24 hours were 98% predictive of eventual outcome. Additional findings highlight the importance of pre-treatment necrotic core, vascular metrics associated with hypertrophic scar formation, and early microvascular changes necessary in both tumorous and peri-tumorous regions to ensure treatment success.

## Introduction

Basal cell carcinoma (BCC) is the most common slow-growing locally invasive malignant epidermal skin tumor, with majority of the lesions localized on the head and neck^1^. There are many different classification schemes for the BCC types and subtypes including nodular, superficial, infundibulocystic, fibroepithelial and the aggressive growth variants such as infiltrative, micronodular and morpheaform (sclerosing) plus some combinations^2,3^. BCC treatment management starts with accurate diagnosis, including histopathology^4,5^ and the development of reliable and non-invasive techniques for its diagnosis is crucial. Currently such non-invasive technologies have limited reliability and more development and validation work is needed^6^. Dermatoscopy is a useful tool in the preoperative prediction of the BCC subtype^7^. However, the evidence of its clinical diagnostic efficacy is somewhat limited and in ambiguous lesions, the BCC subtype has to be assessed histopathologically^8^.

Modern optical imaging modalities such as wide-field fluorescence imaging^9^, confocal microscopy^10^, optical coherence tomography (OCT)^11–13^, polarization-sensitive OCT^14,15^, OCT-based angiography (OCA)^16,17^ and photoacoustic imaging^18^ and their combinations^19,20^ offer new possibilities for detection and visualization of human skin tumor structure and microvascular network in real time. OCT is particularly promising as it offers non-invasive structural and functional 3D imaging of biological tissues with high spatial resolution^11^, in particular for BCC ^9,13,21^. For the first time in this study, we used spectral domain OCT/OCA system to assess the after-treatment effects following photodynamic therapy (PDT) in patients with different BCC subtypes.

PDT involves administration of photosensitizes which preferentially accumulate in malignant tissues, followed by local light activation at appropriate wavelength, producing oxygen free radicals and singlet oxygen^22^. The generation of these reactive oxygen species subsequently causes tumor cell death by apoptosis, necrosis and autophagy through cellular oxidation, damage to the microvasculature, induction of a local inflammatory reaction and immunological system activation^23^. In addition to tumor eradication, PDT’s particular appeal for BCC management is due to its excellent cosmetic outcome in treating superficial lesions^24^, although for nodular BCC the efficacy may be hindered by light penetration limitations. Moreover, usually these treatments are performed with topical application of aminolevulinic acid (ALA), which must diffuse through the skin, thus limiting the Protoporphyrin IX production in deeper layers of the tumor^25^. This study used i.v. administration of the photosensitizer Photodithazine, a glucosamine salt of chlorine (e6), which is clinically approved in Russia and Belarus^26–28^. Photodithazine is also attracting interest of researches in other countries for pre-clinical laboratory experiments^29,30^ and for microbial inactivation, showing promising results^31,32^.

It is a well-known fact that PDT success relies on three fundamental components – photosensitizer, light, and oxygen – all of which may influence the effectiveness of the treatment. To ensure that each of these three ‘ingredients’ is present in the appropriate amounts for PDT to succeed, various quantitative measurements have been attempted^33–37^. An “explicit” approach to PDT dosimetry whereby some combination of these three important variables is measured may be most direct. Singlet oxygen luminescence monitoring is an example of an “explicit” approach for PDT dosimetry^33–36^. Alternatives such as indirect or “implicit” PDT dosimetry methods have also been studied, with photosensitizer photobleaching being the most common. High levels of photobleaching are generally correlated with strong photodynamic response^38–40^, but there are important exceptions^41,42^. Another “implicit” approach for PDT dosimetry is based on measuring the vascular treatment response, as visualized by microvascular OCT (Doppler^43^, speckle-variance^44^ or OCA^41,45^) or photoacoustic imaging^18^. The advantages and limitations of various PDT monitoring approaches have been examined^18,39–43,46^.

OCA used in this study could be promising for clinical use, enabling noninvasive diagnosis and then treatment monitoring during and after PDT of BCC patients. Prior work has shown the feasibility of clinical OCT monitoring for measuring PDT-induced structural tissue changes in skin cancer^47^. However, since many lesions appear quite homogeneous on structural OCT, the utility of such studies for PDT monitoring may be limited. It is also well known that during and shortly after PDT, the vascular damage play an important role in the tumor response^22^. OCA technologies can potentially provide a convenient, practical, and information-rich imaging platform to assess PDT response at the early stages after the treatment. The purpose of this study was thus to assess blood microcirculation in the different BCC subtypes and peri-tumoral regions prior to, shortly after (0 and 24 hours) and long-term (3, 6 and 12 months) post-PDT treatments.

## Results

### Pre-PDT BCC subtypes assessment

In the first part of this study, tumor and surrounding normal skin vasculature were imaged in all patients. A total of 93 pre-PDT OCA data sets were acquired from the 31 lesions of our 27-patients cohort. Of these, 78 suitable-quality *en-face* OCA data sets were selected for quantitative analysis and categorized into nodular, superficial, sclerosing, and nodular-with-necrosis BCC subtypes. 15 data sets had to be rejected due to motion artifacts, as this would affect vascular metrics quantification. The results presented below indicate that it is useful to single out a fourth subtype: nodular-with-necrotic-core BCC. All these subtypes have specifics in their histological structure and angiogenesis, and may exhibit different responses to PDT which is the main focus of this study. Differences in response are likely related to variations in oxygen and nutrients requirements, local perfusion details as well as photosensitizer and light distributions.

**Figure 1:**
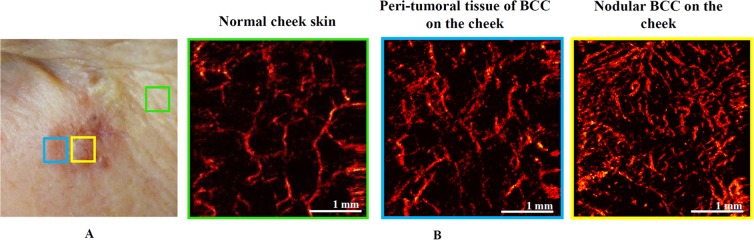
OCA of normal skin, peri-tumoral region and BCC lesion itself prior to PDT. (**A**) Skin photograph showing the locations of OCA imaging: normal facial cheek skin distant from the tumor and treatment field (green square), visually inflamed peri-tumoral region (blue square) that was included in the 5-mm margin illumination field, and BCC tumor center (yellow square). (**B**) Corresponding *en-face* OCA images, showing increasing density and disordered microvascular network in the progression from normal to tumorous tissue.

Figure 1 shows typical microvascular networks for normal cheek skin, visually inflamed peri-tumoral tissue around the visible BCC lesion, and central BCC regions. OCA of the BCC exhibits a dense network with irregular shape and diameter of blood vessels that are significantly different from the normal skin. Overall this figure presents qualitative visual evidence that different microvascular features in normal skin and BCC lesions are present, and OCA is capable of their visualization as has previously been reported^19^.

**Figure 2:**
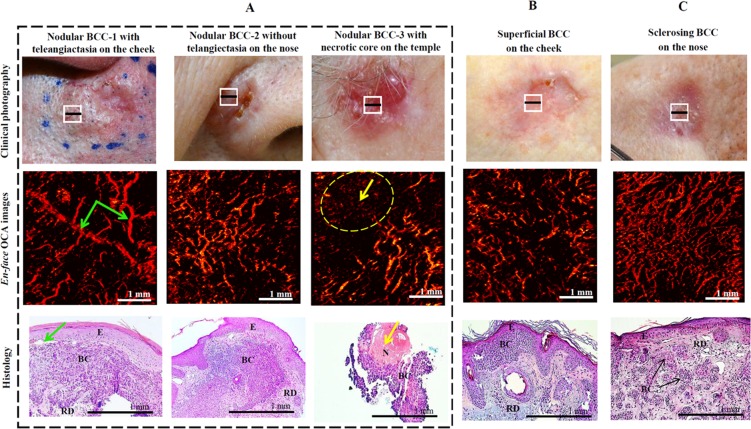
The most common primary BCC subtypes as seen on *en-face* OCA images, demonstrating the spectrum varying from sparse thick vessels (green arrows) to thin vessels with high vascular density. (**A**) Nodular BCC-1 with telangiectasia, same BCC-2 without telangiectasia, and BCC-3 with necrotic core region (black block); (**B**) superficial BCC; (**C**) sclerosing BCC. *En-face* OCA images were taken in regions indicated by white squares in clinical photographs. Histology images were taken in regions indicated by black lines. The yellow arrows in OCA (dotted oval) and histology images indicate necrotic regions (N). Histologically, BCC is characterized by a proliferation of atypical basaloid cells (BC) in the reticular dermis (RD) parallel to the epidermal (E) surface.

Figure 2 shows typical OCA images of the three BCC subtypes – nodular, superficial and sclerosing – taken from different facial skin lesions; corresponding white light photographs and H&E histology images are also included. In addition, nodular-with-necrosis BCC is introduced in the current paper as a separate 4^th^ subtype due to the peculiarity of its microvascular network and its atypical PDT response (discussed below). Nodular BCC is the most prevalent subtype on the face (Fig. 2A). The nodular BCC-1 (left column) includes large nests of basaloid cells in the reticular dermis as detected histologically (lower row). Shiny nodule with a smooth surface may have telangiectasia with distinct “tree-like” branching (dilated capillaries with a diameter of 0.1–1.0 mm), and OCA image confirms the presence of characteristically enlarged branching microvascular networks. Another case of nodular BCC-2 (middle column) shows an increasingly irregular vascular distribution with thinner vessels more densely organized and lacking the large dilated vessels characteristic of telangiectasia. Further, some nodular BCC-3 subtype (right column) presents with necrotic core without ulceration, where some regions exhibit denser vascular network (lower right of its OCA *en face* projection) whereas others regions appear essentially perfusion-free (upper left of its OCA image). The lack of vascular supply limits the intravenous delivery of the photosensitizer, resulting in a poor PDT response. Histologically superficial BCC is characterized by a proliferation of atypical basaloid cells parallel to the epidermal surface (Fig. 2B). The corresponding OCA shows thin and thick branching vessels with increasingly irregular vascular distributions. Finally, histologically sclerosing BCC is characterized by accumulation of basaloid cells in a densely fibrous stroma (Fig. 2C). The corresponding OCA images reveal a denser vascular network when compared to the nodular and superficial subtypes.

Progressing beyond the descriptive qualitative observations of Figs. 1 and 2, we now seek to quantify OCA’s ability to distinguish among different BBC subtypes as well as visually normal skin. In particular, we focus on two prognostically important categories for guided biopsy and optimal treatment choice – (1) differentiating BCC lesions from normal skin, and (2) differentiating BCC subtypes with necrotic core from other BCC lesions.

**Figure 3:**
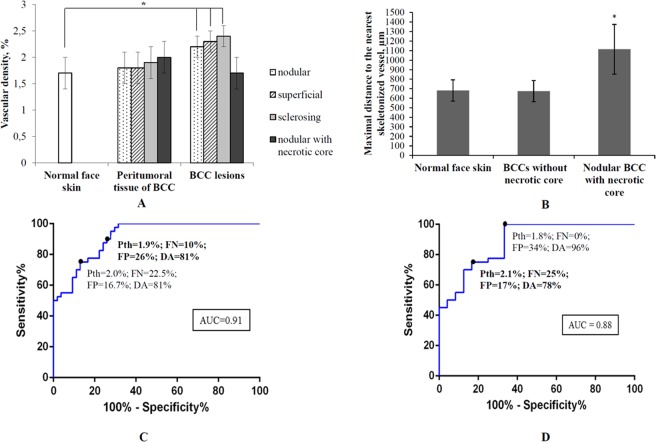
Quantitative assessment of pre-PDT vascular density in normal skin, peri-tumoral BCC tissues and the four (nodular, nodular with necrotic core, superficial and sclerosing) BCC subtypes. (**A**) Averaged vascular density results for the various regions derived from *en-face* OCA images; asterisk (*) indicates a statistically significant difference between normal skin and nodular without necrotic core, superficial and sclerosing BCC subtypes (Bonferroni post-hoc test for multiple comparisons, with p < 0.001); double asterisk (**) indicates a statistically significant difference between nodular BCC with necrotic core from nodular without necrotic core, superficial and sclerosing BCC subtypes (p < 0.001). (**B**) Maximal distance to the nearest skeletonized vessel results for the various BCC subtypes derived from *en-face* OCA images; asterisk (*) indicates a statistically significant difference between nodular BCC without necrotic core and normal skin and all the other BCC subtypes (Bonferroni post-hoc test for multiple comparisons, with p < 0.001). The results are shown as mean ± SD. (**C**) ROC for differentiating normal face skin from nodular, superficial and sclerosing BCC subtypes based on OCA’s vascular density metric. (**D**) ROC for differentiating the nodular-with-necrotic-core BCC (more clinically challenging pathology) from the more treatable superficial, sclerosing and nodular subtypes based on OCA’s vascular density metric. Dots on the curve represent possible choices of the OCA vascular threshold value (P*th*), yielding the listed false negative (FN), false positive (FP) and diagnostic accuracy (DA) values. The curves look stepwise because of limited sample size. The two sets of values on each curve represent possible choices of the vascular density threshold which provide similar diagnostic accuracies but different percentage of false positive and false negative outcomes. Bold font numbers indicate the choices of the vascular density P*th* using a trade-off between the percentages of the FN and FP outcomes and the greatest DA. Additional abbreviations: ROC - receiver operating characteristic; AUC - area under ROC curve.

The vascular density as calculated from OCA images was chosen as a relatively simple, robust and informative quantitative metric to enable both (1) and (2). For problem (1), Fig. 3A illustrates statistically significant differences in the vascular density for nodular, superficial and sclerosing BCC subtypes, compared to normal skin (Bonferroni post-hoc test, p < 0.05 indicated by * on the plot). Despite the similar average vascular density in normal skin and in nodular-with-necrotic core BCC (Fig. 3A), they can be readily differentiated by the level of uniformity. To assess the uniformity for each pixel, we calculated the distance to the nearest skeletonized vessel in each OCA images (Fig. 3B). The maximum value for such distance for the entire image generally occurs in the largest vessel-free area. It also can be considered as characteristic size of the vessel-free area, at least when it is surrounded by vessels on all sides rather than being on the edge or corner of the image. Larger vessels-free areas can be seen in BCC tumors with necrosis, compared to more uniformly distributed vascular network in case of normal skin or BCC without necrotic core. For differentiation problem (2), we further note that the average value of vascular density from nodular-with-necrotic-core BCC subtypes differed significantly from the other three BCC subtypes (Bonferroni post-hoc test, p < 0.001; indicated by ** on the plot). Peri-tumoral BCC tissues of all four subtypes yield intermediate values of the vascular density (middle histogram cluster).

The choice of the OCA diagnostic parameter (vascular density value) for the differentiation of (1): any BBC subtype from normal skin, and (2): nodular with necrosis BCC from the three other BCC subtypes is a trade-off between the FN and FP rates. There is no clear guidance in the peer-reviewed literature or standard of care documents about such trade-off and the shown thresholds are somewhat arbitrary based on authors’ clinical experience. For differentiation between normal skin and all analyzed BCC subtypes vascular density threshold equal to 1.9% was proposed. This threshold provides FN = 10% and FP = 26% (81% diagnostic accuracy (DA)). The area under the ROC curve (AUC) was equal to 0.91.

Similarly to Fig. 3C,D shows the ROC for differentiating between nodular BCC with necrotic core (more challenging subtype for PDT treatment) from superficial, sclerosing and nodular BCCs. The OCA vascular threshold value (Pth) was chosen at to 2.1%, resulting FN = 25%, FP = 17%, DA = 78%. The area under the ROC curve (AUC) was equal to 0.88.

With the chosen vascular density thresholds, the BCC from normal skin identification is 81% accurate and differentiation between nodular-with-necrotic-core BCC and superficial, sclerosing and nodular subtypes is 78% accurate. This is important with the understanding that lack of vascular supply limits the intravenous delivery of the photosensitizer, resulting in a poor PDT response.

### OCA monitoring of BCC PDT treatments

**Figure 4:**
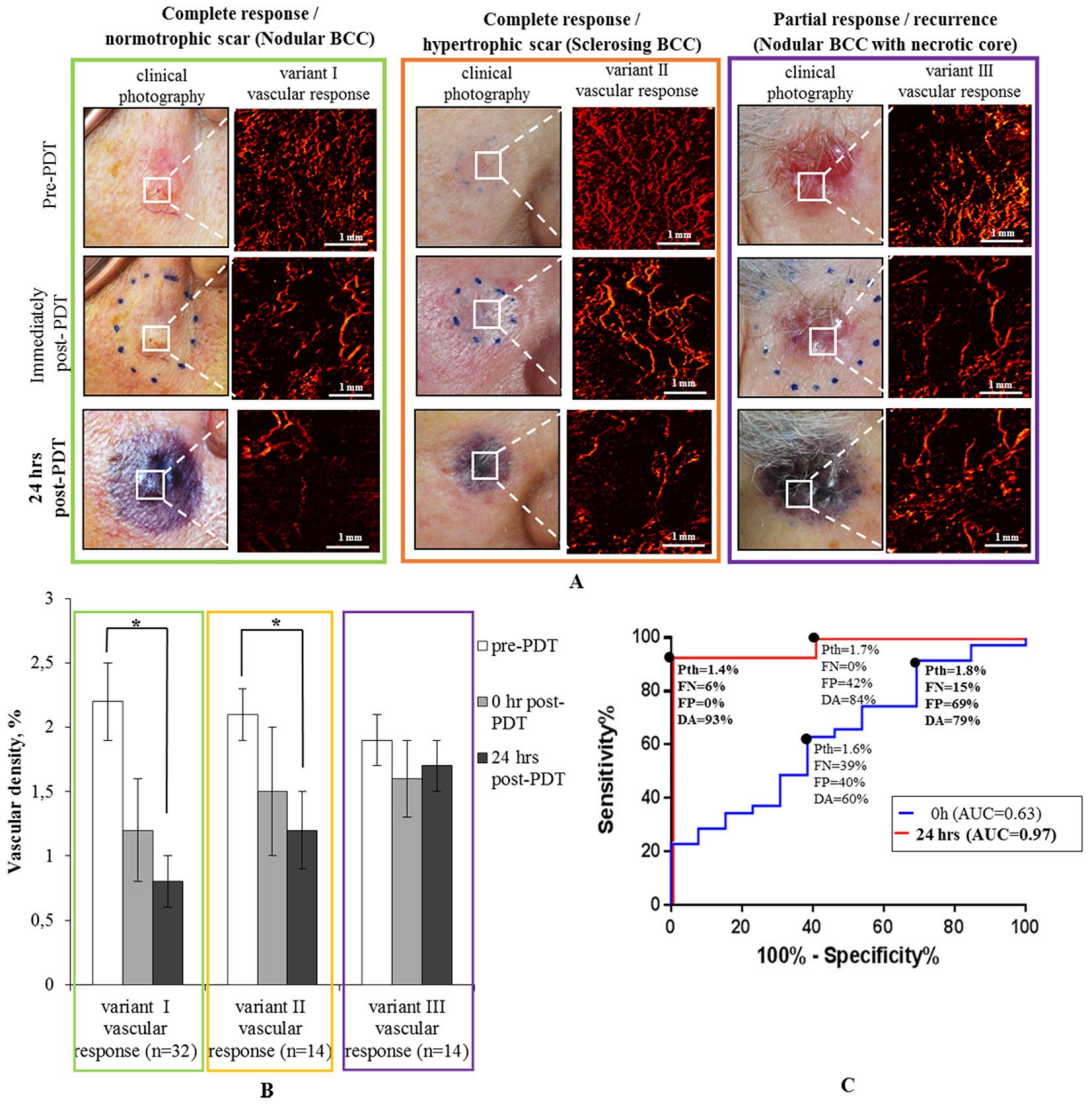
Three variants of short-term PDT vascular response in the BCC lesion, illustrating different clinical and cosmetic outcomes with complete and partial responses. (**A**) Clinical photographs of lesions with corresponding representative OCA *en-face* vascular patterns at pre-, 0 h and 24 hrs post-PDT that yield complete and partial responses. (**B**) Corresponding quantification of the three variants of vascular responses in (**A**) based on OCA’s vascular density metric. The results are shown as mean ± SD. Asterisk (*) denotes a statistically significant difference between vascular density at 24 hrs post-PDT compared to pre-treatment levels, with p < 0.001 (Student’s t-test); n = number of analyzed OCA image data sets in each cohort. (**C**) ROCs for differentiating between complete PDT responders (variants I, II) and recurrent BCC (variant III - partial response) based on short-term post-treatment OCA monitoring results. Dots on the curve represent possible choices of the OCA vascular threshold value (P*th*), yielding the performance metrics (FP, FN and DA) listed below each P*th*. Bold font numbers indicate two possible threshold choices for the vascular density P*th*.

**Figure 5:**
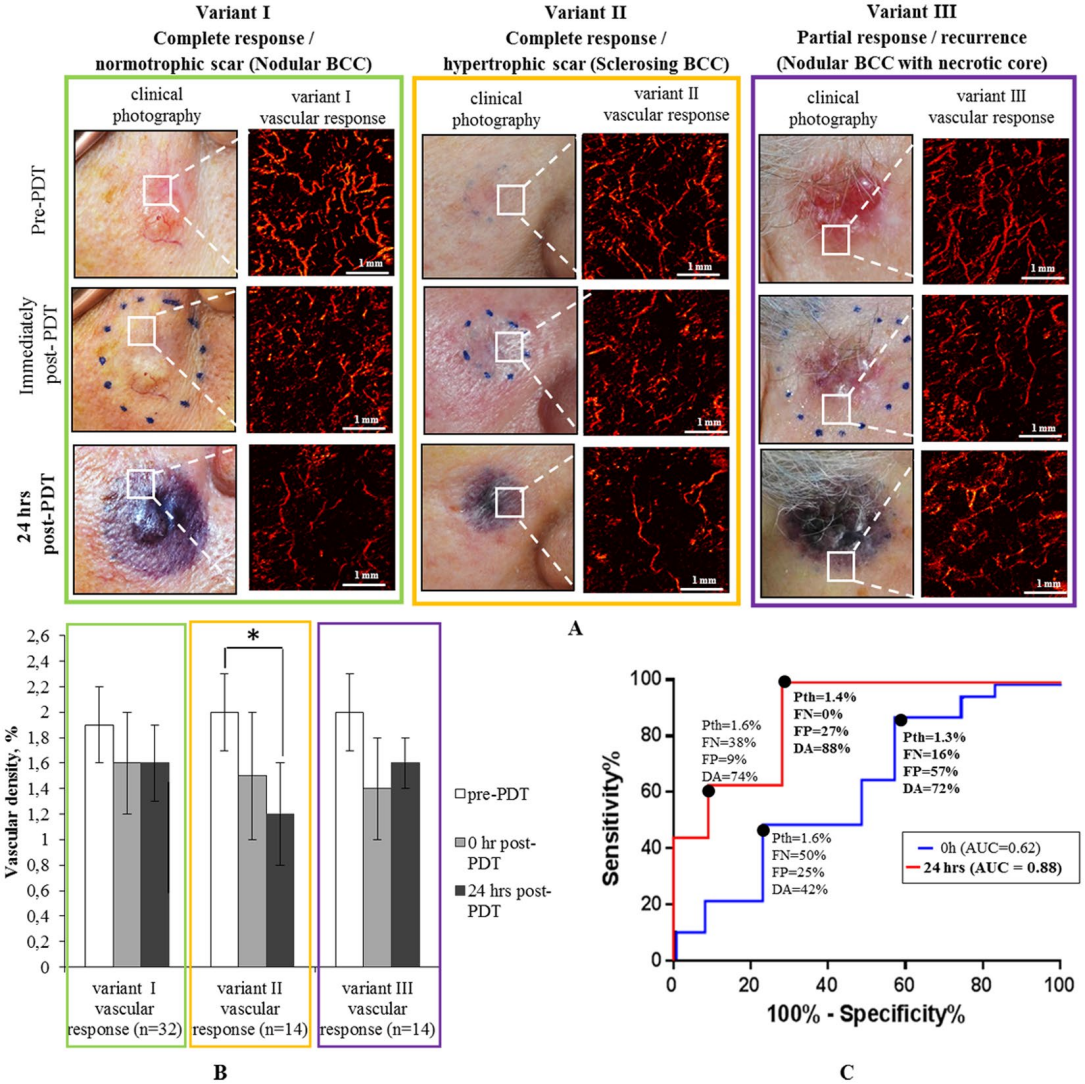
Three variants of short-term PDT vascular responses in the peri-tumoral tissue of BCC, yielding different clinical and cosmetic outcomes with complete and partial responses. (**A**) Clinical photographs of lesions with corresponding representative OCA *en-face* vascular patterns at pre-, 0 h and 24 hrs post-PDT that yield complete and partial responses. (**B**) Corresponding quantification of the three variants of vascular responses in (**A**) based on OCA’s vascular density metric. The results are shown as mean ± SD. Asterisk (*) denotes a statistically significant difference between vascular density at 24 hrs post-PDT compared to pre-treatment levels, with p < 0.001 (Student’s t-test); n = number of analyzed OCA image data sets in each cohort. (**C**) ROCs for differentiating between complete PDT responders (variant II) and complete PDT responders (variant I) for prediction hypertrophic scar formation based on short-term post PDT OCA monitoring results. Dots on the curve represent possible choices of the OCA vascular threshold value (P*th*), yielding the performance metrics (FP, FN and DA) listed below each P*th*. Bold numbers indicate the choices of the vascular density P*th* where diagnostic accuracy is maximized.

In the PDT monitoring context, the vascular damage in center of the tumor and in the peri-tumoral irradiated tissues was monitored within 24 hours (Figs. 4 and 5) and at later times (3, 6 and 12 months) post-PDT. In the long-term follow up, all BCC subtypes resulted in three types of clinical/cosmetic outcomes: I – complete response with normotrophic scar (green box in Figs. 4 and 5), II – complete response with hypertrophic scar (orange box in Figs. 4 and 5), and III – partial response (purple box in Figs. 4 and 5).

Figure 4 shows representative images and quantitative OCA analysis, demonstrating a spectrum of different vascular and clinical responses in the center of BCC tumors within 0 and 24 hours after PDT. Immediately post PDT (t = 0 h), vascular damage was detected in all variants but in variant I and II it was the most pronounced (Fig. 4B**)**. The percent of remaining (visible = non damaged) functional vessels post-PDT was 1.2 ± 0.4% (~56% relative to pre-PDT), and minimum reaction was found in partial responders with 1.6 ± 0.3% of functional vessels (~83% relative to pre-PDT). At 24 hours post-PDT, the vascular density continued to decrease <50% compared to pre-treatment levels for the tumors with complete response, with p < 0.001 (Fig. 4B). Specifically, the tumor vascular density decreased to 0.8 ± 0.2% in case of variant I (~37% remaining functional vessels relative to pre-PDT) and 1.2 ± 0.3% in case of variant II (~56% remaining functional vessels relative to pre-PDT). For recurrent BCC (variant III – partial response), the vascular density 24hrs post-PDT reduced only to 1.7 ± 0.2% (~88% remaining functional vessels relative to pre-PDT) (Fig. 4C).

Figure 4C illustrates the t = 0 h and t = 24 hrs post-PDT ROCs for differentiating complete and partial responders. Not surprisingly, the ROCs clearly show that the vascular state 24h after the treatment (AUC = 0.97) was more effective in predicting the treatment response when compared to the immediately-post treatment OCA results (AUC = 0.63). As seen in Fig. 4C, setting the threshold value to 1.4% on the 24-hr-post ROC curve yields DA = 93% with FP = 6% and FN = 0% for BCC recurrence prediction as assessed 12 months post-PDT.

The additional OCA imaging of the vascular response in the peri-tumoral tissue of BCC within 0 and 24 hours after PDT also can predict the PDT cosmetic outcome, specifically the type of scar. To illustrate this possibility, Fig. 5 shows that the vascular response in the BCC peri-tumoral tissue 24 hours after PDT was able to predict hypertrophic scar formation 12 months post-PDT. As seen from Fig. 5C, setting the OCA vascular density threshold to 1.4% on the 24-hr-post ROC curve yields DA = 88% with FP = 0% and FN = 27% for BCC hypertrophic scar formation prediction during 12-month post-PDT.

**Figure 6:**
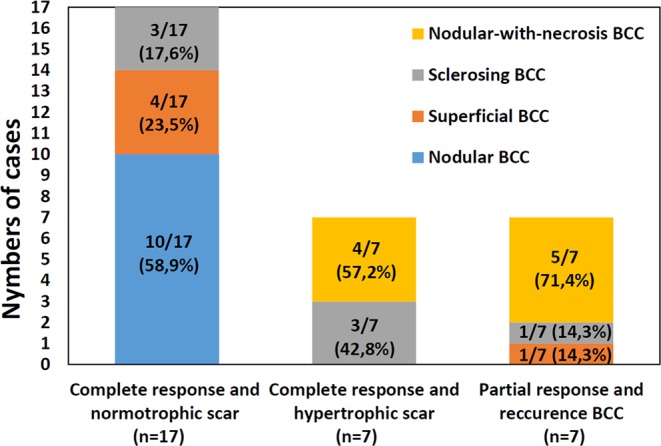
Summary of clinical and cosmetic PDT outcomes for all four BCC subtypes at the end of 12 months follow-up; n = number of analyzed BCC lesions in each cohort.

The 12 months follow-up treatment outcome for different BCC subtypes revealed importance of the OCA for differentiation of new subtype – nodular BCC with the necrotic core. It is clear that nodular BCC with the necrotic core is least appropriate for the PDT (72% of the partial response) as it did not show complete response with normotrophic scar formation. Hypertrophic scars with slower healing were mostly observed in the sclerosing and nodular with necrosis BCC subtypes. The normotrophic scar has mostly observed in the superficial and nodular BCC subtypes (Fig. 6).

**Figure 7:**
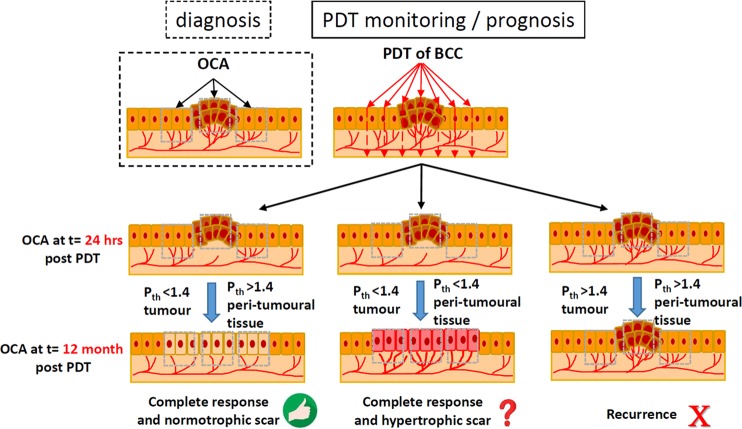
OCA-based PDT clinical and cosmetic outcomes assessment scheme, based on tumor and peri-tumorous microvascular reaction in 24 hours post therapy. P*th* - threshold value choices of the OCA vascular response.

To summarize, a simple-to-implement prediction protocol based on OCA visualization at 24 hours post PDT can be proposed. Overall, tumor vascular reaction correlates with clinical outcome (complete response/recurrence) in 12 month post PDT, while peri-tumorous vascular reaction correlates with cosmetic outcome (normotrophic/hypertrophic scar). If the average density of perfused vessels inside the tumor does not exceed 1.4% on OCA images, it is a good predictor of complete response to PDT. Otherwise recurrence is more likely. If overall density of perfused vessels in the peri-tumorous irradiation tissues is less than 1.4% on OCA images, PDT is likely to lead to the hypertrophic scar formation. These considerations are graphically summarized in the flow chart of Fig. 7. In this study, the three most commonly known subtypes of BCC – nodular, superficial and sclerosing – were examined. Additionally, in view of the above-mentioned significant differences in the reaction to PDT, a fourth subtype was introduced: nodular-with-necrotic-core BCC.

## Discussion and Conclusions

In this paper, OCA yielded better results for distinguishing BCC from normal skin, and for detection of nodular-with-necrotic-core BCC subtype, compared to conventional structural B-mode OCT images. OCA technology is capable of real time 3D perfusion visualization, and represents a promising tool for BCC management for diagnosis, prognosis and treatment response follow-up. Previous studies using OCT-based angiography could only delineate lesion presence^16,17^ without BCC subtypes differentiation. This paper describes OCA assessment of four BCC subtypes - superficial, nodular, sclerosing and nodular-with-necrotic-core. The last subtype is introduced in the current paper and normally not considered as a separate subtype. This can facilitate more precise biopsy location and determination of the necrotic region, as well as better prediction of the PDT outcome based on such pre-treatment information.

The main functional features visualized on OCA imaging, such as changes in vascular vessel density occurring within 24 hours in tumors post-PDT, were quantified to predict the treatment outcome for different BCC subtypes. As previously reported by Hasan *et al*.^18^ and Sirotkina *et al*.^41,45^ in preclinical models, vascular-targeted PDT can effectively destroy the tumor vasculature 24 h after the treatment. In this clinical study at 24 hours post-PDT a decrease in vascular density to 1.4% and less on OCA images compared pre-PDT level was found for complete responders. This is likely because photosensitizer used here is not vascular-targeted, implying both cellular and vascular damage for this PDT regimen (2 hour interval from systemic injection to light irradiation). This decrease in vascular density was larger and statistically significantly different from the smaller vascular density decrease with more than 1.4% on OCA images for non-responders that exhibited BCC recurrence. Overall, for the studied vasculature-targeted PDT with i.v. photosensitizer administration, OCA-based vascular density 24 hours after PDT was able to predict the 12 months recurrence with 93% diagnostic accuracy. Furthermore, we believe that the selective sensitivity of OCA to perfused vessels can also be useful for tumor response assessment in (more commonly used) topical PDT. Regardless of the possible differences in the vascular damage mechanisms, the resultant blood flow interruption can be readily detected by OCA. But since the vascular damage itself – mechanism, severity, manifestation, etc – will likely depend on the relevant PDT and imaging details, for example drug application to light irradiation interval and post-treatment OCA time, additional treatment-specific optimization studies will be needed.

The PDT response for the more treatable superficial and nodular BCC subtypes^48^ is compared with more challenging sclerosing and nodular-with-necrosis subtypes^49^. Sclerosing subtype is not commonly treated with PDT because of the high degree of invasion^50^. However, the sclerosing BCC patients in our study were all over 75 years old, and the common treatments – surgery and radiation therapy – had high risks of side effects. Thus, PDT was deemed as the preferable therapy option, also taking into account that the BCC thickness was less than 5 mm for those patients. Furthermore, Fig. 6 illustrates that such cases are indeed treatable with no recurrence and normotrophic or hypertrophic scar formation. The PDT applicability to nodular-with-necrosis BCC is questionable –50% of the BCC is recurring in 12 months follow up and all non-recurring cases have hypertrophic scar.

A common PDT protocol for BCC therapy involves the topical application of ALA; unlike the intravenously-administered photosensitizer used in this study, ALA diffuses through the tumor and generates the PpIX which is the actual photosensitizer. Concerning the corresponding methylaminolaevulinate (MAL)-PDT treatments, frequent recurrences for nodular BCC cases have been reported in the literature^51–53^. Such poor results are likely due to the deep location of this type of BCC, so that the topically applied photosensitizer does not penetrate sufficiently. In contrast, in our study *all cases* of the nodular BCC were successfully treated. This is probably due to the improved access of the photosensitizer to the tumor by intravenous administration and the intrinsic high PDT efficiency of Photodithazine that combines strong anti-angiogenic and direct cell damage actions. To the best of our knowledge, that there is only published report about the prevalence of the nodular “ulcerated” BCC subtype, with 20% MAL-PDT application^39^. However, in this study there are only 10 nodular-with-necrosis BCC, and only 6 out of them had a necrotic core without superficial ulceration. This limited sample size suggests that such necrotic core before treatment may exist in almost half of the nodular BCC cases. Such necrotic core could be related to already poor blood supply in the area before the treatment. In these cases, it may be inappropriate to perform PDT to further reduce blood supply. Instead the PDT should be more focused on direct cellular phototoxicity, which requires a different treatment regime^39^. In particular, a combination of vascular and topical delivery of the photosensitizer could improve the treatment response.

Nodular-with-necrosis BCC heals much slower and often ends up with the hypertrophic scars. The post-op vascular density could be helpful to decide whether or not to perform a second PDT procedure to minimize recurrence. It was previously shown that the early detection of partial responses after MAL-PDT could improve the patient’s management, in particular reduce treatment area and number of treatments^54^.

Furthermore, we show that differences in vascular density in the peri-tumoral tissue of BCC post-PDT may be helpful for better prediction of the cosmetic outcome and types of scar formation. OCA based vascular density assessment 24 hours after PDT in the peri-tumoral tissue were able to predict formation of hypertrophic scars with an accuracy rate of 88%. It may be helpful to plan additional therapy to improve treatment response and scar quality.

In longer-term follow up post-PDT when scar formation and connective tissue remodeling is occurring (3, 6 and 12 months), OCA detects different recovery and increasing vascular density. Normotrophic scars with faster healing at 3–6 months and decrease in the vascular density 12 months follow-up post-PDT were mostly observed in the nodular and superficial BCC subtypes. Hypertrophic scars with slower healing and significant increase of vascular density at 12 months follow-up post-PDT were mostly observed in the sclerosing and nodular-with-necrotic-core BCC subtypes. Such hypertrophic scar formation is a common complication of wound healing^55^. The scarring process associated with PDT likely makes the recognition of partial responses and/or recurrences clinically more difficult. PDT induces a dense sclerosis involving the superficial dermis with a sharp lateral delimitation^46^. Importantly, reaction of PDT-treated but visually normal skin around the BCC margins was not a good predictor of healing response.

Recurrences represent the main challenge regarding BCC management, and the development of non-invasive techniques for tumor diagnosis, progression assessment, and response monitoring is critical. OCA demonstrated promising capability to diagnose and assess tumor progression, and monitor therapy after-effects. It also showed that vascular-targeted PDT has poor response in nodular-with-necrotic-core BCC, which informs clinical choices for different PDT protocols in those cases. Moreover, OCA may become an important tool for implicit PDT dosimetry, being able to predict long-term BCC response as early as 24 hours after the treatment. This information may prove relevant for the clinician, to help decide on performing an additional PDT session in order to improve the treatment response.

## Materials and Methods

### Patients

The study was approved by the Institutional Review Board of the Privolzhsky Research Medical University and the Nizhny Novgorod Oncology Clinic. Informed consent was obtained from all participants and/or their legal guardians enrolled in the study. All methods were performed in accordance with the relevant guidelines and regulations. The study included twenty-seven patients (7 males and 20 females, age 50–82) with 31 histopathologically confirmed localized BCC lesions not deeper than 5 mm as measured by ultrasound. All patients underwent fluorescence imaging to monitor the presence/verify sufficient accumulation of photosensitizer in the tumor prior to PDT. The tumor diameter range was 0.8–2.5 cm. The lesions were mostly facial, located on the cheek (n = 14), forehead (n = 5), temple (n = 6) and nasal area (n = 6). All lesions were histopathologically classified into nodular (n = 10), superficial (n = 4), sclerosing (n = 7) subtypes of BCC and a nodular-with-necrotic-core (n = 10) BCC subtype was considered separately. During the studies we closely interacted with practicing dermatologists who confirmed the lesion classification.

Additionally, normal regions of facial skin (cheek, forehead, temples and nose) were studied (n = 27). The follow-up period was restricted to <12 months. The cases of suspected insufficient PDT efficacy and thus possible recurrence were confirmed by biopsy in the 3–12 months interval post PDT. The OCA vascular status before and at various time points after the treatment within a year were collected and analyzed, for correlation with lesion response and cosmetic outcome.

### Histopathology

All lesions were biopsied and examined histopathologically. Histological slides were stained with hematoxylin and eosin (H&E). Histopathological analysis was performed by a single experienced pathologist (SSK).

### Photodynamic therapy

PDT was performed at two hours after intravenous injection of 1.0 mg/kg Photodithazine (Glucamine-salt of chlorine E6 approved for clinical PDT, VETA-GRAND, Russia). The light-drug interval was chosen based on the Photodithazine pharmacokinetics information (clearance from the body typically occurs within 24–48 hours) provided by the manufacturer^56^. Photodithazine is approved in Russia for clinical treatments of tumors in bronchi, esophagus, skin, and oral cavity^26–28^. Such PDT regimen likely results in a combination of cellular- and vascular-targeting mechanisms of action. Just before the PDT treatment, fluorescence imaging was performed to verify that sufficient drug accumulation occurred in the tumor. Fluorescence imaging of photosensitizer accumulation was performed using a commercial camera-based imaging system (Fluovizor; Atkus, St. Petersburg, Russia) with 660 nm LED excitation (bandwidth 20 nm) that coincides with the absorption band of the PS. 775 nm band pass emission filter (SL 755/90; Photooptic-filters, Obninsk, Russia) rejects the excitation LED radiation and enables detection of the longer wavelength fluorescence from 710 to 800 nm. Acceptable fluorescent contrast ratio between the tumor and BCC-unaffected normal tissue was at least 1.3 and reached in all patients. In the paper^57^, the contrast ratio <1.3 was consider to be “low accumulation”. It was demonstrated that PDT in case of low photosensitizer accumulation lead to recurrence.

The tumors were then irradiated by a laser (Lakhta-Milon; Milon Laser, Moscow, Russia) emitting at 662 nm, laser spot size was in the range 1.8–3.5 cm depending on the tumor diameter, incident irradiance = 280–310 mW/cm^2^, total fluence 150 J/cm^2^ ^26^. The optical power was verified before each procedure. A 5-mm irradiation margin was added around the tumor periphery, to treat visually normal tissue, possibly including the tumor-feeding vasculature; previous work has demonstrated that PDT cure rates drop when ‘normal’ tissue around the tumor was not irradiated. The irradiation for each patient’s BCC + 5-mm margin contour was delivered through a conformally-shaped cutout in an opaque material.

### OCT-based angiography imaging

A spectral domain (SD) OCT system operating at 1.3 μm central wavelength with axial resolution of ~10 μm in air and lateral resolution of ~15 μm with an imaging speed of 20 kA-scans/sec was used^23,58^. Infrared laser power incident on tissue was low (~2 mW), and the imaging sessions were relatively fast (26 seconds total scanning time). Microvasculature visualization was based on temporal speckle variations rate of full complex signal with high-pass filtering of B-scans comprised of highly overlapping A-scans, as per M-mode-like OCT^59–62^. Optically linearized spectrometer reduced signal computational complexity and allowed real time visualization of angiographic images^63^. 3D OCA images were represented as 2D maximum intensity projections (MIPs), showing the vascular network *en-face* over the entire visualization depth. The scanned volume was 3.4 × 3.4 × 1.5 mm.

Importantly for this clinical pilot study, the OCA technique is non-invasive and enables label-free imaging of perfused blood flow, which is crucial to detect because shortly after PDT the blood flow is already blocked. Previously, we have carefully verified OCA’s ability to correctly visualize perfused vessels by comparing the OCA microvascular images with the fluorescent dye (FITC) visualized vessels on confocal microscopy in murine tumor models (CT26)^41,45^. As shown, same perfused vessels were visualized on both OCA and FITC contrasted images prior to PDT; at post-PDT stasis, these vessels consistently disappeared from both images.

**Figure 8:**
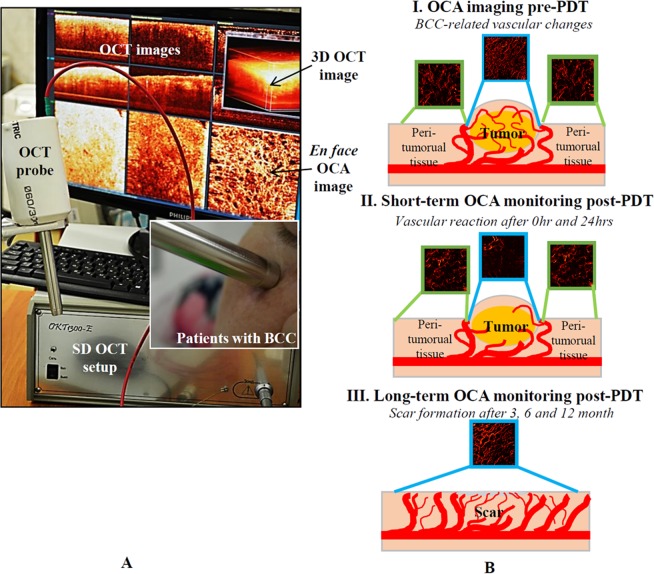
(**A**) Spectral domain OCT imager for clinical monitoring of patient’s microstructure and microvasculature. The optical probe was placed in gentle contact with the skin surface using an articulated arm, with real-time image display on the system’s monitor. (**B**) Summary scheme of OCA assessment of PDT success.

The forward-viewing OCT optical probe (8 mm diameter, length 10 cm; lateral scanning enabled by galvo-controlled beam steering with the probe housing^64^) was positioned on sitting subject’s facial skin with gentle contact using an articulated arm apparatus (Fig. 8A).

### OCA processing

For OCT angiographic analysis and quantification, the 3D microvascular network imaging was converted to 2D *en-face* images, with MIPs showing the vascular network *en-face* over the entire visualization depth of ~1.5 mm. For angiographic analysis and quantification, each OCA image was binarized and skeletonized^65^. The nearest skeletonized vessel was defined as a pixel on the skeleton line closest to the given image pixel. The search of the nearest neighbor from the given subset (vessel skeleton in our case) was done with *k-d tree* algorithm^66^, for which the calculation time logarithmically depends on the number of points. Many quantitative metrics of the resultant microvascular networks are possible^67^ for this study, a relatively simple and robust one proved sufficient for BCC sub-classification and PDT monitoring. Specifically, a vascular density metric was calculated as the ratio of the total number of skeletonized (vasculature) pixels in the analyzed image area to the total number of pixels in this area. In addition to the vascular density ratio, vascular heterogeneity was assessed by calculating the maximal distance of each tissue pixel to the nearest skeletonized vessel in each image. In the case of the large avascular heterogeneous regions, this value is expected to be larger than in case of uniformly distributed more homogeneous vascular networks.

OCA images were acquired from three locations: (1) tumor center, (2) visually normal skin peri-tumoral that was included in the 5-mm margin illumination field, and (3) normal facial skin distant from the tumor and treatment field. Post-PDT imaging was performed immediately (t = 0 hours), t = 24 hours and t = 3, 6 and 12 months (the latter including the presence of treatment scar) (Fig. 8B).

### Statistical analysis

The variable for statistical inter-group comparison was the vascular density calculated from *en -face* OCA images. The data distribution normality was verified using the Kolmogorov–Smirnov criterion, justifying the selection of the T-test metric. Descriptive statistics results are expressed as mean ± SD. Since this study includes comparison of multiple groups, a Bonferroni post-hoc test for multiple comparison test was selected, to compare (1) normal skin to three BCC subtypes (nodular, superficial and sclerosing), and (2) nodular-with-necrotic-core BCC subtype to nodular, superficial and sclerosing BCC. Further, the paired 2-sided Student’s T-test was used to compare BCC lesions pre-PDT to 24 hrs post-PDT, and similar comparison was made for peri-tumoral tissues. In all cases, the differences were considered statistically significant when p < 0.05.

The statistical data processing was done in MS Excel 2010 with a public domain software plugin for statistical analysis STATISTICA 10 (StatSoft Inc, Tulsa, Oklahoma, USA). The ROC-related calculations of the sensitivity, specificity, diagnostic accuracy and area under the ROC were performed with Prism v6 statistical software (GraphPad Software, La Jolla, California, USA).
